# mHealth 2.0: Experiences, Possibilities, and Perspectives

**DOI:** 10.2196/mhealth.3328

**Published:** 2014-05-16

**Authors:** Stefan Becker, Talya Miron-Shatz, Nikolaus Schumacher, Johann Krocza, Clarissa Diamantidis, Urs-Vito Albrecht

**Affiliations:** ^1^Institute for Drug SafetyDepartment of NephrologyUniversity Hospital EssenEssenGermany; ^2^Marketing DepartmentOno Academic CollegeKiryat OnoIsrael; ^3^Lifepatch GmbHKasselGermany; ^4^Black Tusk AGFilzmoosAustria; ^5^Division of NephrologyUniversity of Maryland School of MedicineBaltimore, MDUnited States; ^6^PL Reichertz Institute for Medical InformaticsHannover Medical SchoolHannoverGermany

**Keywords:** mHealth, mobile applications, text-messaging, stakeholders, consumer, mobile technology, technology interoperability, behavior change, regulation

## Abstract

With more than 1 billion users having access to mobile broadband Internet and a rapidly growing mobile app market, all stakeholders involved have high hopes that this technology may improve health care. Expectations range from overcoming structural barriers to access in low-income countries to more effective, interactive treatment of chronic conditions. Before medical health practice supported by mobile devices ("mHealth") can scale up, a number of challenges need to be adequately addressed. From a psychological perspective, high attrition rates, digital divide of society, and intellectual capabilities of the users are key issues when implementing such technologies. Furthermore, apps addressing behavior change often lack a comprehensive concept, which is essential for an ongoing impact. From a clinical point of view, there is insufficient evidence to allow scaling up of mHealth interventions. In addition, new concepts are required to assess the efficacy and efficiency of interventions. Regarding technology interoperability, open standards and low-energy wireless protocols appear to be vital for successful implementation. There is an ongoing discussion in how far health care-related apps require a conformity assessment and how to best communicate quality standards to consumers. "Apps Peer-Review" and standard reporting via an "App synopsis" appear to be promising approaches to increase transparency for end users. With respect to development, more emphasis must be placed on context analysis to identify what generic functions of mobile information technology best meet the needs of stakeholders involved. Hence, interdisciplinary alliances and collaborative strategies are vital to achieve sustainable growth for "mHealth 2.0," the next generation mobile technology to support patient care.

##  Introduction

**Figure 1:**
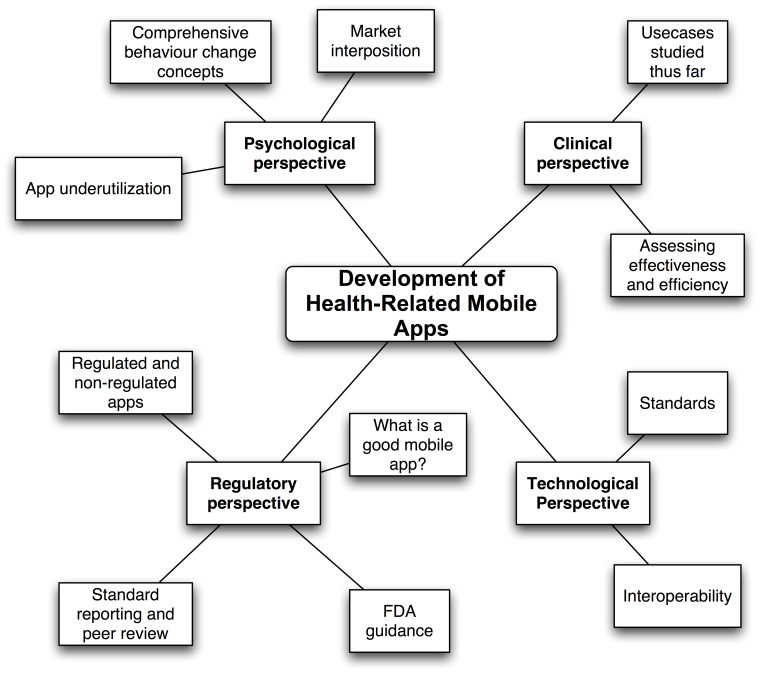
Development of health-related mobile applications and necessary viewpoints.

With more than 6 billion mobile phone subscribers, it is estimated that 75% of the world population has access to mobile communication [1]. The number of devices with broadband capabilities increased to more than 1 billion worldwide [2]. Associated with the advances in hardware is the rapid evolution of a mobile app industry: with the promise “There's an app for that,” Apple introduced its App Store in 2008. From conception, the number of mobile apps offered increased from 500 to 850,000, with more than 50 billion recent app downloads [3]. New mobile technology offers novel system solutions for a variety of needs in daily life. Consumers, patients, providers of medical services, software developers, governments, and non-governmental organizations are excited about the opportunities mobile communication technology is likely to offer in terms of improving access to health care and delivery, engagement of patients, and clinical outcomes [4]. Whereas an estimated 63% of adults who own a mobile phone use their devices for Internet access, approximately 34% of them use their phones as their primary means for going online [5]. The availability of mobile Web access is allowing individuals who may not have broadband capabilities readily available in their home to access Internet content. With more than 97,000 health-related mobile applications and approximately 1000 new apps being published every month, there are high hopes in the market, which is expected to grow by more than 25% per year [6]. What is termed “mobile Health” or “mHealth,” broadly defined as medical or public health practice supported by mobile devices [4], encompasses a variety of contexts: use of mobile phones to improve point of service data collection, care delivery, patient communication, use of alternative wireless devices for real-time medication monitoring, and adherence support [7]. These new services are developed within a colorful new industry, which brings together a variety of disciplines sometimes lacking understanding of each other’s perspective. This viewpoint paper gathers experiences, evidence, and prognosis in the mHealth market from a psychological, medical, technological, and regulatory perspective (Figure 1). The idea of this paper was conceived at the Medicine 2.0 Conference 2013 in London, where 3 of the authors (TMS, UVA, and SB) met. They thought that a wider scope was required to fully understand the context of mHealth and started to write this paper, which evolved to be an interesting learning process for all contributors.

## Psychological Perspective: What Are Consumers’ Needs and Expectations?

### Overview

An entire continuum of health care needs can be addressed via apps, broadly in two main areas: changing consumer/patient behavior in health-related areas (eg, diet and exercise) and improving the implementations of prescribed treatment regimens. As apps proliferate, psychological questions arise—to what degrees are apps not only downloaded but also used, and what can improve their uptake and effectiveness? This section will examine these questions in terms of consumers' needs and expectations about health-related apps.

### App Underutilization

A study undertaken independently by the IMS Institute for Healthcare Informatics analyzed 43,689 mHealth apps, and suggested that despite an enormous number of health care apps available for download, most apps are underutilized [8]. The study found a significant skew in download volume for health care apps, with more than 50% of available apps achieving fewer than 500 downloads. Indeed, 5 apps account for 15% of all downloads in the health care category. The study clearly demonstrates that, to date, most efforts in app development have been in the overall wellness category with diet and exercise apps accounting for the majority of available apps. Despite the large number of health care apps developed so far, the majority has only simple functionality and does little more than provide information. In addition, studies show a high attrition rate for Internet interventions and mobile applications [9,10], which may be a reflection of an early interest in the novelty of the application, with a decline in eagerness as the novelty of the intervention wears off. Reasons for the limited number of downloads as well as hurdles for improved uptake can be found for all stakeholders. Patients currently face an overwhelming array of health care apps to choose from, with little guidance on quality or support from their doctors. A commendable effort was made by Happtique, a subsidiary of the Greater New York Hospital Association’s for-profit arm GNYHA Ventures, to certify apps such as “amazing abs,” “CalorieCounterPro,” and “ControlMyWeight,” providing some guidance through the app jungle. Yet the certification program was recently suspended because 2 certified apps were shown to threaten data privacy [11]. Data privacy and patients’ ability to alert and defend themselves against privacy breaches have been discussed in the context of electronic health records [12]. These concerns are increasingly relevant as apps encourage consumers to pour potentially sensitive health data into them. Furthermore, apps developed to date do not completely cover the areas of health care responsible for the largest expenditures; for example, patients facing multiple chronic diseases and those typically over the age of 65. Elderly patients are likely to be among the top health care spenders but smartphone penetration is lowest among this group, with only 18% of the US population using them, compared to 55% of those aged 45-54 years.

### How Apps Can Influence Behavior Change

A recent study that analyzed the written descriptions that developers provide with 3336 paid health and fitness apps in Apple's iTunes store identified 3 main psychological factors that can drive behavior change. These are (1) predisposing, which increase the user’s capability; (2) enabling, which facilitates an authentic experience for users; and (3) reinforcing. These 3 factors assist the user in establishing and strengthening relationships and performing the required actions repeatedly [13].

Most of the apps were coded as either predisposing or enabling with only 6.65% of apps classed as reinforcing. Only 1.86% (62/3336) of apps included all 3 factors, which may help explain why health behaviors have not shifted dramatically since the emergence of apps. Another issue raised by the authors is that some health topics appear to have fewer apps associated with them. Examples of relatively neglected app topics include substance abuse, mental and emotional health, violence prevention and safety, and sexual and reproductive health. This lack of coverage of all health and well-being topics reduces the degree to which a person can take control of his or her health using apps alone. Interestingly, the more expensive apps (cost greater than $0.99) were identified as more credible or trustworthy, more recommendable to clients in a professional setting, and more likely designed to promote health and prevent disease. This reinforces the point that the more expensive apps are more likely to be based on theory leading to behavior change.

### Market Interposition

In a review of apps and their potential [14], the authors marvel at the combination of phone and tracking systems, which years ago was considered science fiction. Yet even they acknowledge that apps “are so rapidly developed that they may or may not meet anyone's needs or expectations.” Indeed, the fact that apps for taking care of various medical conditions are so readily available, and that this availability does not require the mediation of a health care professional, marks what Roth has dubbed “marketplace interposition” [15]. “Marketplace interposition” is where technological advancement encourages society to tacitly permit self-treatment and unauthorized practice of medicine through consumer access and actual use. What remains to be examined is whether and how people actually use this new right to self-treatment to take care of their health. Roth goes on to suggest that “marketplace interposition” means that it is the commercial marketplace that is effectively rejecting the federal prohibition against self-treatment and rejecting state prohibition of the unauthorized practice of law. “Marketplace interposition” is real and cannot be ignored if practical discussions are to be had regarding the appropriate level of regulation in mHealth. Society wants access and demands mobility. Some commentators argue for less regulation for certain diagnostic apps that presuppose a person is receiving medical attention. The reality is that the risk of injury is higher for lay people operating without physician oversight; some measure of regulation is necessary to ensure safety.

### Potential Limitations of Apps From the Psychological Perspective

A recent review of social media interventions designed for health improvement has found that the effectiveness of such interventions is enhanced when they are combined with an off-line, not just virtual, social encounter [16]. This suggests that users of an app might also benefit from real-life support and interaction around the health issue they are managing digitally. Another limitation pertains to human cognition in general. Whenever information is presented, especially in probabilistic format, there is a chance that approximately half the people who read it will not be able to understand what it means. For example, women who received information about the BRCA 1/2 gene mutation, which is associated with a high prevalence of breast and ovarian cancer, misunderstood the risk magnitude associated with it [17]. Similarly, men who were asked about a gene that is associated with prostate cancer showed considerable difficulty in understanding the related risk probabilities [18], which may reflect a lack of tailoring to individuals with limited health literacy and numeracy. Probabilities are not the only issue to be taken into account when designing apps for optimum comprehension and effectiveness. Because approximately 90 million Americans read at or below the level of a sixth grader, it cannot be assumed that the entire population has sufficient literacy skills for coping with medical apps [19]. Comprehension issues may also arise among physicians when using apps, or when explaining/recommending them to patients. This can in part be tied in with the amount of information the patient/consumer/doctor has to read and assess. In an experiment that examined residents and medical students, the ability to choose the right course of action was diminished when participants were presented with 10 or 20 options as opposed to 3 [20]. Thus, even though digital media offers limitless possibilities for information presentation, the amount of information might be a liability, not an advantage. Even when no right answer exists, choice overload can be debilitating. This applies to the digital world, too. For example, “Switch to Health” was a company that offered incentives for physical behavior [21]. The company originally offered points that could be redeemed for an award from around 70 awards, ranging from an iTunes download to a Wii. However, the company later decided to categorize the awards so that consumers did not have to analyze all 70 of them, potentially being discouraged in the process. Comprehension is a key to the success of apps, but is not necessarily sufficient. For consumers to act upon information, they need to trust its source. Trust cannot merely be assumed, as has become apparent from parents' lack of adherence to the US Food and Drug Administration (FDA)/UK Medicines and Healthcare products Regulatory Agency warning regarding the administration of cough and cold medication for children. In a study examining parents’ trust of the medical system and its representatives, parental trust was highest for their pediatricians, and then for the pharmacists. However, it was substantially lower for the health system [22-24]. Further research is required to assess to what degree people trust their app and its recommendation, and whether this drives health outcomes. So far, apps have been referred to as a homogenous entity. Yet not all apps, it appears, are created equal in terms of potential effectiveness, or are as accessible to consumers in terms of cost. Higher quality apps, ones that are based more on theoretical content, most notably the health belief model, are more expensive [25]. This may be a barrier for successful uptake in a target population. It has been suggested that future collaborations between behavior change experts and app developers could foster apps that would possibly lead to better health outcomes.

One final point of limitation is that of “marketplace interposition,” where the consumer is to assume responsibility over his or her health. However, the availability of a good, relevant, and efficient app does not guarantee that it will be used by patients, let alone by patients who need it the most. A hint of the potential issues and potential self-selectiveness of patients comes from a study showing that smokers are at an increased risk for prostate cancer, yet less likely than nonsmokers or previous smokers to seek testing [18]. The creation of effective apps alone is not sufficient for improving health; thus, app development requires the close involvement of public health and clinical professionals who can directly speak to the health problems to be addressed and the functional requirements.

### Clinical Perspective: Is mHealth Technology Effective in Patient Care?

Use of mobile technology in patient care may be attractive for two reasons: One is its magical appeal for those interested in global public health to solve one of the most difficult problems facing global health efforts—that of structural barriers to access [26]. Another is its promise in better meeting patients’ needs and offering more effective and efficient health care services. Particularly in chronic disease lifestyle changes, drug adherence and vital sign monitoring play essential roles in therapy management [27]. The intriguing question is whether mobile computing offers effective and efficient system solutions for these requirements. Currently, mHealth interventions do not have sufficient evidence, which would allow a scale up beyond pilot studies. Systematic reviews on the topic by Free and colleagues found that while multiple studies have been conducted, many are of poor quality and very few have found clinically significant benefits of the interventions [28,29]. It seems as though providers using mobile communication technology to connect with their patients achieve an improved overall patient-provider communication [30]. Authors studying short message service (SMS; text message)-based appointment reminders showed a statistically significant but modest increase in patient attendance compared to no reminders [28,31,32]. However, text message reminders were no more effective than postal or phone call reminders and texting reminders to patients who persistently missed appointments did not significantly change the number of cancelled appointments [28]. Particularly in chronic disease (eg, cardiovascular disease, chronic obstructive pulmonary disease, HIV infection, chronic kidney disease, and diabetes), lifestyle changes are a key component of the therapeutic management to reduce morbidity and mortality [33,34]. In their meta-analysis, Free et al identified 75 controlled trials (studies that compare the outcomes of people who do and do not receive an intervention) of mobile technology-based health interventions delivered to health care consumers that met their predefined criteria [29]. Twenty-six trials investigated the use of mobile technologies to change health behaviors, 59 investigated their use in disease management, most were of low quality, and nearly all were undertaken in high-income countries [29]. In 1 high-quality trial that used text messages to improve adherence to antiretroviral therapy among HIV-positive patients in Kenya, the intervention significantly reduced the patients’ viral load but did not significantly reduce mortality [35]. In 2 high-quality UK trials, a smoking intervention based on text messaging (txt2stop) more than doubled biochemically verified smoking cessation [36,37]. Other lower-quality trials indicated that using text messages to encourage physical activity improved diabetes control [38-41]. Combined diet and physical activity text messaging interventions also had no effect on weight, whereas interventions for other conditions showed suggestive benefits in some but not all cases [41].

### Lessons Learned for Future Projects From the Clinical Perspective

On setting up a mobile communication intervention, one needs to bear in mind that next to the above-mentioned psychological factors, text messaging is more likely to work under a set of ideal parameters to include [26,42] the presence of follow-up; messages that are highly relevant in frequency, wording, and content; and personally tailored interventions.

The need for improved health is apparent. Chronic disease is on the rise, and some attribute it to factors that cannot easily be changed, certainly not with digital tools, such as increasingly busy lifestyles, unhealthy eating habits, and a highly competitive workplace [43]. On this theme, it has been proposed that collaboration between patients and physicians, one that takes into account limited time resources through a remote health-monitoring service, may provide an end-to-end solution. The goals for such a service, which would be operated via a mobile device, collect data, for example, blood pressure readings or weight from the patient through a mobile phone, provide these data to doctors through a Web interface, and enable doctors to manage the chronic condition by providing feedback to the patients remotely. Whether such systems will be effective and efficient is difficult to say. So far, evidence on telemonitoring (eg, hypertension and cardiac failure) is not allowing a general implementation for all patients [44-47]. It will be interesting to see whether mobile applications in the hands of patients will facilitate telemonitoring of chronic disease and allow a more widespread deployment. Finally, despite the aforementioned evidence, most mHealth interventions can be seen as the equivalent of black boxes [26]: The problem of pilot studies in text messaging is that a particular style of a black box application is compared to a situation without any black box application. The question for future trials is what generic functions of mobile technology are being deployed by users. In this context, further randomized controlled trials are necessary. Novel research designs such as data farming in cooperation with service providers may increase the evidence base and offer new insights regarding how to best implement such new tools.

## Technological Perspective: Anything Goes?

### Overview

Driven by the rapidly changing innovation cycles in the telehealth solution environment, it seems that everything can be solved relatively quickly. Many press releases give the unrealistic impression to the public audience that everything is possible. If there is the perception that an app is an “independent piece,” this is wrong.

### App Development

From a technological perspective, a mobile application is the result of an interweaving chain of many hardware and software components. The complexity is increasing with each additional hardware component and each new software release. The “Continua Healthcare Alliance” with their standardization and certification processes may help attenuate this phenomenon [48]. In addition to overall complexity, limited power supply remains an additional concern. For this reason, long-term mobile wireless monitoring is currently not feasible. In addition, software development and maintenance faces the problem of ever-increasing potential variations in end-customer apps. There is a cascade of at least 4 large operating systems for smart devices, nonstandardized drivers, and protocols customized by different mobile phone producers, and programming and design techniques, resulting in an exponential number of configurations for apps. When developing for telemonitoring, there is a choice of several new, partially mutually exclusive wireless-standards: Bluetooth Low Energy, ANT+, NFC, and ZigBee.

The level of support of these standards by mobile phone producers and operating systems is quite heterogeneous, reaching from “not at all” to “partially working” to “full support.” The older and much better implemented standards such as Bluetooth 2.0 or WiFi have high energy consumption and are electromagnetically problematic in hospitals, airplanes, etc. Even within one operating system, pressure to update is high: Customers are urged ever increasingly to allow auto-update on their smart devices. Declining auto-updates often results in malfunctions of apps or even the basic functions of a mobile phone or tablet. Changes in operating system generations can be quite drastic, as Microsoft demonstrated with its change from Windows 7 to Windows 8. Even though Windows 7 apps still run on Windows 8 computers, they do not have the same look and feel as the new apps and have to be started from the old desktop, which Microsoft originally wanted to remove completely but had to re-introduce because of pressure from users groups. Furthermore, Windows 7 applications cannot access some of the new hardware such as Bluetooth Low Energy. Another example is the market leader for mobile devices, Android, where at least 5 layers of software affect the function on an app (eg, for heart rate-monitoring): the hardware drivers for phone components, the official Android version, the customized Android components, the wireless protocols, and design and software components.

Each of these layers can change independently and may force the others to follow, and for every new combination, an app update may become necessary [49]. Apart from increasing costs, this poses a significant problem for the development of medical devices: According to regulations, any change in medical software automatically leads to a new certification process [50,51]. This is further aggravated by the installation of additional or new versions of apps on smart devices, sometimes not even voluntarily but rather caused by automatic updates running in the background. These newly installed apps may influence the medical app, so that a medical application running on a mobile phone would actually have to be recertified several times a week. Some producers have therefore branded their own versions of operating systems, albeit with relatively high costs, restricting automated updates and installation of foreign apps. Apart from the logistics effort this implies, it also frustrates end users because they either have to accept a separate device for their medical app or sacrifice the personal freedom of their mobile phone. If a producer decides to ignore regulatory requirements of recertification necessitated by updates, he does this at his own risk, with potentially unproportionally expensive lawsuits involved. In combination with rapid development cycles, the resulting maintenance and development costs have had a noticeable negative influence on business cases [52]. A positive aspect and hope for future products is the availability of new instruments for producers and developers to roll out and maintain their software products [51]. Nowadays, all mainstream operating systems feature their own app stores that include easy-to-use mechanisms for auto-updating. Software frameworks for apps support unit testing. Low-energy wireless protocols seem to reach a standard and are implemented on more and more devices. By conforming to these standards, and by prioritizing the opportune planning of test environments, many of the foreseeable problems resulting from updates can be mitigated.

### Technological Challenges

So far, mHealth is evolving as a patchwork of incompatible applications that are not interoperable [53]. Mobile devices, medical hardware, and health information often cannot communicate with each other and share data. There is good evidence that improved therapeutic management can be achieved through an integrated health systems strengthening approach [54]. One may expect that enabling mHealth systems to share information with one another as well as with broader eHealth systems can increase efficacy and efficiency of patient care and reduce cost associated with data collection. International collaboration between industry and research institutions is vital for developing standards that are approved by all stakeholders. This demand for “interoperable systems” is going along hand in hand with the aspect of “open standards” [53]. The latter are crucial for equity in eHealth and mHealth. As discussed on the World Health Organization forum on data standards, there is a danger that closed standards may create a knowledge barrier for developers in low- and middle-income countries [55,56]. There are authors discussing the need for a governing body, for example, the World Health Organization, to certify such open standards and enable countries’ access to standards that meet key criteria [57].

##  Legal and Regulatory Perspective: How Can We Make Medical Apps Trustworthy?

### Overview

Transparency is a vital aspect for users and customers of a market that has low entry barriers and is flooded by mobile apps. Authorities are called on to develop regulatory instruments to ensure users’ safety, which on the on the other hand may slow down technological and scientific developments.

### A Good Mobile App

As mentioned before, being easily accessible and highly available makes smart devices, such as smartphones and tablet personal computers very attractive for both private and professional areas of application. Nevertheless, there are certain professions in which special care must be taken when making use of such devices and the medical field is one of them. In a medical context, when mobile smart devices are used in combination with add-ons that are connected either directly or via some wireless technology, for example containing additional sensors for fulfilling their purpose, such as for measuring blood glucose levels, manufacturers already have to conform to the usual laws and regulations that are in place for medical devices, although depending on the jurisdiction, they may or may not be well adapted to the specifics of mobile devices. Usually, regulation does not only encompass hardware-based add-ons but also extends to an app running on the smart device. When used in such a combination, the need for regulation may be easily obvious and once conformance has been proven, may indicate a certain level of trustworthiness for users. Still, standalone smart devices and the apps running on them may also pose a significant threat to a patient’s safety and privacy if the necessary safety measures are not observed. A more casual user might not even notice flaws and shortcomings of the mobile device itself or its apps, although there are a number of potential problems to keep in mind. Among others, issues may range from not all promised functionality being available to outright miscalculations, erroneous or incomplete content, technical deficiencies, and other usage restrictions. In addition, because nonprofessional users of medical apps often do not have sufficient backgrounds, they may often be unable to judge whether the information they are being confronted with is correct. In a worst-case scenario, an app might give a user a false sense of security, thereby keeping him from seeking medical advice and help in a timely manner. One such example was shown in a study about apps that make use of the camera functionality of mobile devices to judge whether skin lesions are suspicious [58]. With the exception of 1 app that simply provided a means to upload the acquired image data to (remote) professionals for evaluation, all other apps (that were based on automatic image analysis) did not have an acceptable recognition rate. If such an app falsely gives an “all clear,” a patient with a malignant lesion such as melanoma might not seek medical help in time and might thus have considerably lower chances of being cured. This is only one of many examples of why—despite all recent advances in the area of mHealth technology as well as health apps and medical apps—appropriate measures must be taken to ensure that such apps conform to necessary standards.

Regardless of their purpose, apps used in a medical context need to be trustworthy, safe, and their user interface must allow efficient use. Especially when apps are used by health care professionals while working on patients, regulatory aspects become relevant because such apps might be subject to applicable medical product laws in the country where they are to be used. The aspect of an “app = medical device” is not trivial because using an uncertified application in a professional setting may lead to legal consequences for both the health care provider as well as his employee, for example, the health care professional who uses the app. Therefore, apps applied in this context also need to follow applicable laws and regulations. In fact, there is a lot of confusion about this topic because manufacturers are often unaware of the fact that their product might be required to undergo the same regulatory processes that are applicable for fever thermometers or cardiac catheters. Nevertheless, it depends on the manufacturer’s declaration of the intended (medical) use of a product whether regulations are applied.

### Regulation

Whether a smart device or app is classified as a medical device depends on its intended use described by the manufacturer. If the intended use matches certain criteria, the manufacturer has to ensure that all appropriate regulations are observed. For the European Market, manufacturers can refer to the MEDDEV Guideline 2.1.1/6 that was published by the European Commission in January 2012 [59]. Although it is legally not binding, it can be quite helpful in interpreting the appropriate European regulation. When it comes to software products, including mobile apps, an application is assigned the label “medical device” if it is intended for diagnostic or therapeutic purposes. Examples may include software that monitors specific parameters of the patient such as the heart rate or other physiological parameters in office settings or even in intensive care, but also software that allows measuring parameters such as the interpedicular or sagittal diameter of the spinal canal from previously acquired image data. It is always necessary for a manufacturer to obtain a CE label for a product falling under the medical device directive before he is allowed to put it on the market. This CE label can only be assigned after the product has successfully undergone appropriate conformity assessment procedures. The details of the regulatory processes vary depending on the risk category to which the product has been assigned. There are similar rules overseas: In the United States, if a device falls under regulation, manufacturers need a premarket notification; a letter of substantial equivalence must be obtained from the FDA. Without this, commercial distribution of such a device is prohibited. The FDA published a guideline aimed at developers of mobile medical apps on September 25, 2013 [49]. Its intent is comparable to the MEDDEV guideline published by the European Commission.

### FDA Guidance on Mobile Medical Applications

The aforementioned guidance document published by the FDA is meant to provide clarification regarding the kinds of products that will warrant a closer look by the FDA in the future. Manufacturers as well as members of the US congress have eagerly awaited the finalized guidelines. In its final form, the guidance document only marginally differs from the draft version that the FDA put up for discussion in July 2011. According to the FDA, 130 comments were taken into account, most of which were affirmative of the draft’s objectives [60].

For the time being, apps that can be classified as medical products but only pose limited risks for their users will not have to undergo regulatory procedures (“enforcement discretion”). The FDA describes this as a “risk-based approach.” This is meant to alleviate concerns about discouraging innovation by putting up regulatory obstacles while still providing adequate protection for consumers. There was only little overt criticism of the guidance document, probably because the FDA refrained from also holding manufacturers of mobile phones and tablets or operators of app stores accountable. Instead, the FDA primarily focuses on apps that convert a conventional mobile device into a medical device or are meant as an accessory to an already regulated medical product. The FDA remains vague about which risk classes apply for apps and accessories and about which exceptions might apply—many interpretations are possible and some uncertainty remains. Because requirements posed on the products depend on the guidelines, the FDA will probably have to integrate clarifications into the guidance document in the near future.

### Nonregulated Apps

Existing laws and regulations are only applicable for a rather small number of apps. The FDA counted only 140 medical apps with FDA approval to date. In addition, governmental regulations only apply to a limited number of apps. There are a number of private certification initiatives [61] for apps that do not have to undergo official regulatory processes, but these usually target professional developers or larger companies with sufficient financial background to pay for certification. Both official regulation processes as well as private certification are often not only expensive but also time-consuming and as such, they are not very attractive, even for those who are willing to conform to the necessary standards.

Although an advantage of private certification initiatives is that they often publish their results quickly, these initiatives often have their own test standards that are usually not disclosed; thus, it often remains unclear how they come to their conclusions. The same holds true for another popular source of information. Although many users rely on articles posted on blogs and other Web pages as well as user comments that can be found directly in the app stores to obtain basic information about an app, the presented information is often questionable because there is often only limited information about the authors’ backgrounds.

Nevertheless, to not gamble away the large potential apps and accompanying mobile devices offer for all kinds of applications, including medicine, providing users with trustworthy apps that are well suited for their purpose is very important. If users were to lose trust in such apps, this might have a negative impact on future sales and hinder future innovations. In addition, one important question remains: How can users be provided with sufficient and trustable information to allow them to judge whether an app meets their needs and whether they can trust it while still keeping cost and effort for distributors and developers at an acceptable level?

### Standard Reporting and Peer Review

The rapid development and distribution processes that are common nowadays do not make it easy for users to assess the safety aspects of using apps in a medical context. One solution with good potential might be to implement a “standard reporting” mechanism for such apps. The idea behind this is to provide manufacturers, developers, and distributors with a comparatively cheap and easy to apply transparent solution for providing potential users with the information they need to form an opinion about an app. This “standard reporting” should encompass all information about an app in a standardized way, which in addition to aiding users, could also simplify comparisons between similar apps and further discussions about the apps whenever desired or could even serve to support peer review processes. The main intent of standard reporting remains to provide users with adequate information to let them easily judge whether an app is trustable and meets their needs. To promote the discussion about standard reporting, an app synopsis was developed that makes use of the idea of standardized reporting [62].

### App Synopsis

The app synopsis we developed is meant to serve as a blueprint regarding the way necessary information may be provided. A number of existing projects and initiatives use criteria that are similar to those specified by the “Apps Peer-Review” project instigated by the *Journal of Medical Internet Research* (JMIR) that was launched in 2013 [63]. Many of the aspects included in the proposed app synopsis [62] are also already covered in the JMIR mHealth disclosure form [64]. Most of the existing projects make use of certification processes and/or review processes. Evaluation results are commonly published using specific channels, for example, a Web page provided by the project or in a scientific journal. For casual users who might not be informed about the existence of such projects, this approach has the disadvantage of low visibility of such evaluations. Also, those who are currently in the market of “app testing” come from many different—often commercial—backgrounds and their funding strategies as well as their areas of interest often differ, ranging from taking a look apps for only a single disease to targeting health apps in general. In some cases, it also remains unclear whether commercial interests might have undue influence on the results and which standards are applied while an app is being evaluated for certification. For example, only recently, the Health app certification program Happtique was halted [65] after an independent evaluation of 2 randomly selected apps out of 19 that had previously been certified by Happtique found serious deficits in these apps, for example, unencrypted personal health information and passwords. These diverse structures do not make it easy for consumers to find the information they need and to judge whether results obtained and published by such entities can be trusted.

The app synopsis on the other hand has a slightly different focus. It tries to integrate aspects from various initiatives that deal with Web-based medical content or medical apps, but there are also some notable differences. In contrast to other initiatives that were previously initiated for Web-based medical content (eg, HONcode [66]) and rely on providing users with evaluations of the content by experts or rating organizations (eg, medCERTAIN [67]), the information for the synopsis is being compiled by the manufacturers or developers themselves and is open for scrutiny and discussion by all interested parties, including experts, and patients. Although in parts, the predefined structure of the synopsis makes use of similar criteria for compiling the information, it keeps everything on a basic level that—in addition to answering questions customers commonly have—can also serve as a starting point for additional, independent testing, for example, by helping to identify appropriate test methods or to find suitable experts to help them with their assessment based on the available information. For users, this means that in addition to an expert‘s evaluation that may be valuable, but may also be biased, they can also access unfiltered information provided by the manufacturer to use in addition to any expert opinions they may find. Thus, altogether, they can use both kinds of information to form their opinion.

Another aspect in which the app synopsis differs from approaches that are available for Web content is that, with the progression of technology, new challenges arise and need to be addressed: users often entrust apps running on their personal devices with (potentially confidential) health information that they would hesitate to enter on a Web page. In addition, mobile devices go even further when collecting information: in addition to what users enter, they can also evaluate data acquired via integrated sensors or additional external sensors for diagnostic purposes and to support decision making on health aspects. One problematic example has already been illustrated above for apps that aim at diagnosing skin cancer [58]. This can also lead to a higher level of risk for users: not only do they have to be able to determine whether the information (diagnosis and resulting advice) they are presented with can be trusted, but also whether the app can be trusted with respect to data security and data protection. For apps, this is certainly also being addressed by other initiatives such as the JMIR Apps Peer-Review [63], but, instead of keeping the compiled information in places that might be “out of sight” for casual users, the results of the synopsis are meant to be published at places where users often first try to get information: the respective app stores as well as manufacturer’s home pages. All in all, the provided information can also be used for an unofficial but collaborative evaluation process of all interested parties, for example, patients, doctors, but also competitors; this may also be an additional building block on the path toward informed patients in the information age.

## Conclusions: Collaborative Strategies Vital to Help Sustain Growth in the mHealth Business

Undoubtedly, the use of mobile technologies in a medical context is highly attractive for patients, doctors, and administrative staff as well as researchers, but in part for different reasons. When using mobile devices and apps in a health context, patients usually have convenience in mind but would also like to stay in control of or be better informed about certain aspects, for example, by recording and evaluating data about their health. This is also emphasized by the continuing growth of the “quantified self” movement. On the other hand, on top of medical aspects, doctors, as well as administrators, hope for help with certain administrative headaches and cost savings [2]. Regardless of their backgrounds, all users want apps that they can trust, although what contributes to “trust” can be quite different depending on the user’s perspective: this may range from an app being easy to understand from the user’s point of view, providing sufficient background information, to studies that support an app’s efficacy as appropriate measures being taken with respect to privacy, security, and data protection. Legal and regulatory aspects must also be kept in mind [62], as well as data security. If mHealth technologies and applications are to be widely adopted, vendors must respect all these requirements. Concerning research, numerous studies have been published that are based on researchers’ best guesses about optimal app-based implementations of specific interventions, yet only a few randomized controlled trials have been published that take a look at the overall situation. With respect to development, additional emphasis must be placed on context analysis to identify which generic functions of mobile information technology best meet the needs of involved stakeholders. Hence, interdisciplinary alliances and collaborative strategies are vital for achieving sustainable growth in the field.
